# Health-related quality of life in amyotrophic lateral sclerosis using EQ-5D-5L

**DOI:** 10.1186/s12955-021-01822-9

**Published:** 2021-07-20

**Authors:** Qian-Qian Wei, Yanbing Hou, Yongping Chen, Ruwei Ou, Bei Cao, Lingyu Zhang, Tianmi Yang, Huifang Shang

**Affiliations:** Department of Neurology, Laboratory of Neurodegenerative Disorders, West China Hospital, Sichuan University, No. 37 Guoxue Xiang, Chengdu, 610041 Sichuan China

**Keywords:** Amyotrophic lateral sclerosis, EQ-5D-5L, Health-related quality of life, Non-motor symptoms, Clinical stage

## Abstract

**Background:**

The study aimed to appraise the health-related quality of life (HRQoL) measured by the five-level EuroQol-5 dimensions (EQ-5D-5L) in amyotrophic lateral sclerosis (ALS), and to explore the associations between non-motor symptoms (mood changes, cognitive disturbances and sleep disturbances).

**Methods:**

EQ-5D-5L descriptive scores were converted into a single aggregated “health utility” score. A calibrated visual analog scale (EQ-VAS) was used for self-rating of current health status. Multiple logistic regression analysis was used to explore the factors associated with HRQoL.

**Results:**

Among the 547 enrolled ALS patients who were assessed using EQ-5D-5L, the highest frequency of reported problems was with usual activities (76.7%), followed by self-care (68.8%) and anxiety/depression (62.0%). The median health utility score was 0.78 and the median EQ-VAS score was 70. Clinical factors corresponding to differences in the EQ-5D-5L health utility score included age of onset, onset region, the ALS Functional Rating Scale-Revised (ALSFRS-R) score, and King’s College stages. Patients with depression, anxiety, and poor sleep had lower health utility scores. Patients with excessive daytime sleepiness and rapid eye movement sleep behavior disorder had lower EQ-VAS scores. Multivariate logistic analysis indicated that ALSFRS-R scores, depression, and anxiety were associated with health utility scores. After adjusting other parameters, ALSFRS-R score, stages, and depression were significantly associated with EQ-VAS scores (*P* < 0.05).

**Conclusion:**

This study examined HRQoL in ALS patients using the Chinese version of the EQ-5D-5L scale across different stages of the disease. We found that HRQoL is related to disease severity and to mood disturbances. Management of non-motor symptoms may help improve HRQoL in ALS patients.

**Supplementary Information:**

The online version contains supplementary material available at 10.1186/s12955-021-01822-9.

## Background

Amyotrophic lateral sclerosis (ALS) is a fatal disease caused by progressive degeneration of both upper and lower motor neurons in the brain and spinal cord [1]. Survival time varies tremendously, ranging from several months to more than 10 years, with a median survival between 3 and 5 years after disease onset [2]. In the past decade,many advancements in the understanding of pathogenesis and treatment of ALS have occurred [3].

ALS functional rating scales and health-related quality of life (HRQoL) questionnaires were adopted as the primary or secondary endpoints in many clinical trials [4]. HRQoL declines as the disease progress, and the rate of change correlates with physical, psychological, existential, and support factors [5, 6]. Among the HRQoL questionnaires, the five-level EuroQol five-dimensions (EQ-5D-5L) questionnaire is a standardized, generic HRQoL questionnaire which was developed by the International EuroQol Research Group [7], and generated the EQ-5D-5L value set for China in 2017 [8]. The measure has been widely used in HRQoL studies on neurological diseases (including ALS, Parkinson’s disease and myasthenia gravis), which showed that it generates a generic standardized measure for health status [9–11]. It is also possible to calculate the varied health utility scores when different aggregated EQ-5D-5L value sets are applied.

A prior study found that the health status (HS) of ALS patients before their diagnosis is highly dependent on the perception of upper and lower limb function [12]. Using clinical trial data, a previous study showed that progressively decreased health utility, as measured by EQ-5D, is associated with increased severity of King’s ALS Clinical stages [13]. Additionally, a systematic review suggested that lower HRQoL scores are associated with higher levels of anxiety and depression [14]. However, the associations between HRQoL and other non-motor features, such as sleep impairment, have not been addressed in different patient cohorts.

The first purpose of the current study was to demonstrate the HRQoL of ALS patients as measured by EQ-5D-5L. We also explored the impact of disease-related factors on utility scores, especially non-motor symptoms (moods, and cognitive and sleep disturbances). Additionally, we investigated the determinants of HRQoL in ALS patients, which we hope will improve patient care and enhance medical intervention and therapeutic strategies. We hypothesized that the HRQoL of ALS patients (as measured by the EQ-5D-5L) would be affected by disease-related motor and non-motor symptoms.

## Methods

### Patients and clinical evaluation

Our cross-sectional study was conducted at the tertiary referral center for motor neuron disease in South-West China (Department of Neurology, West China Hospital of Sichuan University, Chengdu, Sichuan province). From May 2018 to May 2020, patients who were diagnosed with definite, probable, or possible ALS (according to the El Escorial revised criteria) were registered in the study [15]. Patients who were classified as possible ALS at the time of registration were reclassified to higher El Escorial levels during the follow-up. Patients with a diagnosis of progressive muscular atrophy, progressive bulbar paralysis, primary lateral sclerosis, and juvenile ALS were excluded. Demographic and disease-related variables were collected. Young-onset was defined as onset age of fewer than 45 years of [16]. Onset forms were classified into spinal (upper or lower limb) and bulbar subgroups. Patients were also divided into two groups according to the ALS Functional Rating Scale-Revised (ALSFRS-R) score [17]. Following the King’s College staging system, ALS staging was based on the presence of symptoms in different nervous system regions– defined as bulbar, upper limb, lower limb, or diaphragmatic [18]. Stage 1 referred to symptom onset or the involvement of the first region. Stage 2A referred to an ALS diagnosis and Stage 2B was defined as functional involvement of a second region [19]. In addition, Stages 4A and 4B were combined and labeled as Stage 4 for analysis.

Executive function was assessed using the frontal assessment battery (FAB) [20]. The Chinese version of Addenbrooke’s Cognitive Examination-revised (ACE-R) was usedto evaluate cognitive function. In line with our previous study, cognitive dysfunction was diagnosed in patients with ACE-R scores lower than 75 [21]. Depression and anxiety were assessed using the Hamilton Depression Rating Scale (HDRS) and the Hamilton Anxiety Rating Scale (HARS). HDRS scores > 7 indicated depression, and HARS scores > 7 indicated anxiety. We evaluated sleep quality using the Pittsburgh Sleep Quality Index (PSQI), the Epworth Sleepiness Scale (ESS), and rapid eye movement sleep behavior disorder Screening Questionnaire (RBDSQ). PSQI scores > 5 indicated poor sleep quality. Excessive daytime sleepiness (EDS) was diagnosed in patients with total a total ESS scores ≥ 10. Rapid eye movement sleep behavioral disorder (RBD) was diagnosed in patients with RBDSQ scores ≥ 5 [22]. Patients were considered to have sleep disturbances based on the results of sleep scales. This study was approved by the institutional ethics committee of West China Hospital [approval No.2015(236)]. All participants provided written informed consents. A brief introduction of the scales used in the present study is shown in Additional file 1: Table 1.

### Assessment of the HRQoL

EQ-5D-5L is a quantitive tool to evaluate health status by measuring perception of questions related to five dimensions of health: Mobility (MO), Self-care (SC), Usual activities (UA), Pain/discomfort (PD), and Anxiety/ depression (AD). Each dimension has five levels: no problems, slight problems, moderate problems, severe problems, and extreme problems. The five EQ-5D-5L descriptive scores may be converted into a single aggregated “health utility” score anchored at 1 (perfect health) and 0 (death). The index values, presented as country-specific value sets, are a major feature of the EQ-5D-5L instrument, facilitating the calculation of quality-adjusted life years that are used to inform economic evaluations of health care interventions. Studies that directly elicit preferences from general population samples to extract value sets for the EQ-5D-5L are completed in several countries, including in China [8]. In our study, the algorithm yielded scores ranging from -0.391 to 1.000, with 0 representing death, 1.000 indicating a state of full health, and negative scores indicating health states worse than death. A calibrated visual analog scale (EQ-VAS) was also used by patients themselves to rate their current overall health status, with endpoints of 100 (best imaginable health state) at the top and 0 (worst imaginable health state) at the bottom. Data were collected via face-to-face interviews, either with the assistance of well-trained neurologists or as self-administrated by literate patients accompanied by a supervisor, to ensure that the questionnaire was completely filled out.

### Statistical analysis

Continuous parameters that were normally distributed were described as the means ± standard deviation (SD). Those with a non-normal distribution were presented as the median values. Continuous variables were compared using Student’s t or Mann–Whitney U tests. One-way analysis of variance (ANOVA) was used to compare variables with three or more groups. Because multiple comparisons were performed, *P* values were Bonferroni-adjusted. Subgroups analyses were conducted according to age of onset, sex, onset region, ALSFRS-R scores, King’s College stages, FAB scores, ACE-R scores, depression, anxiety, EDS, RBD, and PSQI. Spearman’s correlation analyses were performed to detect relationships between EQ-5D-5L values and clinical variables. The correlation coefficient (r) described the correlations in varying degrees. A stepwise multiple logistic regression analysis model was used to explore the potential determinants of HRQoL. The healthy utility score was used as the dependent variable. Patients were divided into two groups according to the median of the healthy utility score and EQ-VAS score. The demographic and clinical variables were included in the regression analysis. The criteria were set at *P* < 0.05 for entry into the model and at *P* > 0.10 for removal of a variable from the model. All b coefficients with 95% CI are presented. A level of *P* < 0.05 was considered statistically significant. All analyses were performed using SPSS 19.0 (SPSS, Inc., Chicago, IL, USA).

## Results

### Patient characteristics

A total of 547 patients were included in the study. Two hundred and seventy-three patients (49.9%) had upper limb onset, 37.7% of patients had lower limb onset, and 12.4% had bulbar onset form. 224 (41.0%) patients were at stage 1, 193 (35.3%) patients were at stage 2, 86 (15.7%) patients were at stage 3, and 44 (8.0%) patients were at stage 4 according to the King’s College staging system. The demographic and clinical features of patients are presented in Table 1. The mean FAB score was 15.8 ± 2.3. The mean ACE-R score was 77.9 ± 13.6. The mean HDRS score was 8.6 ± 6.8. The mean HARS score was 5.8 ± 5.6. The mean PSQI score was 5.2 ± 3.6. The mean ESS score was 5.2 ± 4.5. The mean RBDSQ score was 2.0 ± 1.6. Patients in Stage 4 had swallowing impairments requiring gastrostomies, as well as respiratory declines requiring non-invasive ventilation, which impacted on scales assessment. Thus, these 44 patients were excluded from further analysis.

**Table 1:** Demographic and clinical variables according to King’s college stages

	Total (n = 547)	Stage 1 (n = 224)	Stage 2 (n = 193)	Stage 3 (n = 86)	Stage 4 (n = 44)	*P* value
Age, years (M (SD))	54.7 (11.4)	54.0 (11.0)	55.1 (11.8)	55.1 (12.7)	55.5 (9.5)	0.796
Male (n, (%))	347 (63.4)	153 (68.3)	114 (59.1)	52 (60.5)	28 (63.6)	0.240
Education, years (M (SD))	9.4 (3.6)	9.7 (3.8)	9.1 (3.4)	8.8 (3.4)	9.5 (3.7)	0.081
Age at onset, years (M (SD))	53.4 (11.4)	52.8 (11.1)	53.8 (11.7)	53.9 (12.6)	53.8 (9.5)	0.865
Disease duration, months (M (SD))	15.5 (14.4)	15.1 (14.3)	15.2 (14.3)	14.5 (13.2)	20.7 (16.5)	0.049
Diagnostic delay, months (M (SD))	14.6 (14.0)	14.3 (13.6)	14.3 (14.1)	13.6 (13.1)	20.1 (16.6)	0.034
Onset region (B, UL, LL)	68, 273, 206	30, 109, 85	22, 96, 75	10, 47, 29	6, 21,17	0.967
ALSFRS-R score, (M (SD)	41.4 (4.4)	44.4 (2.4)	40.8 (3.4)	37.6 (4.2)	36.4 (5.1)	< 0.001*
BMI, M (SD)	22.6 (3.1)	22.8 (3.1)	22.5 (3.2)	22.5 (3.1)	22.5 (3.2)	0.806

ALSFRS-R, Amyotrophic Lateral Sclerosis Functional Rating Scale-Revised; B, bulbar onset; UL, upper limb; LL, lower limb; BMI, body mass index; M, mean; SD, standard deviation

^*^Significant difference. *P* values were Bonferroni-adjusted

### EQ-5D-5L screening

**Fig. 1:**
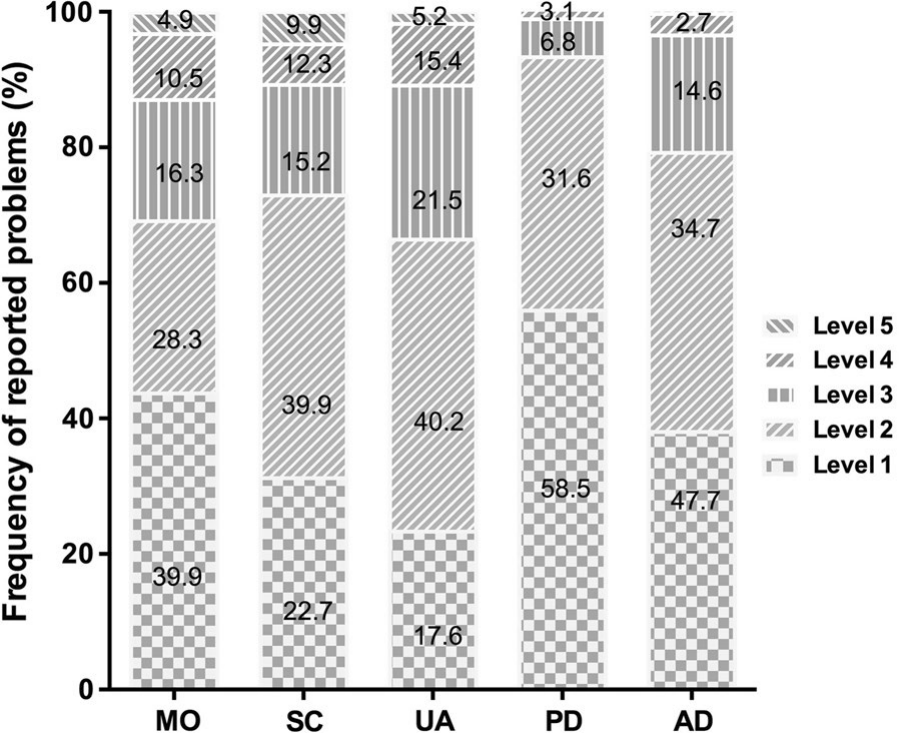
Distribution of health problems in ALS patients across the five dimensions of the EQ-5D-5L. Abbreviations: MO: mobility, SC: self-care, UA: usual activities, PD: pain/discomfort (PD), AD: anxiety/depression. Level 1: no problems, level 2: mild problems, level 3: moderate problems, level 4: severe problems, level 5: extreme problems

**Fig. 2:**
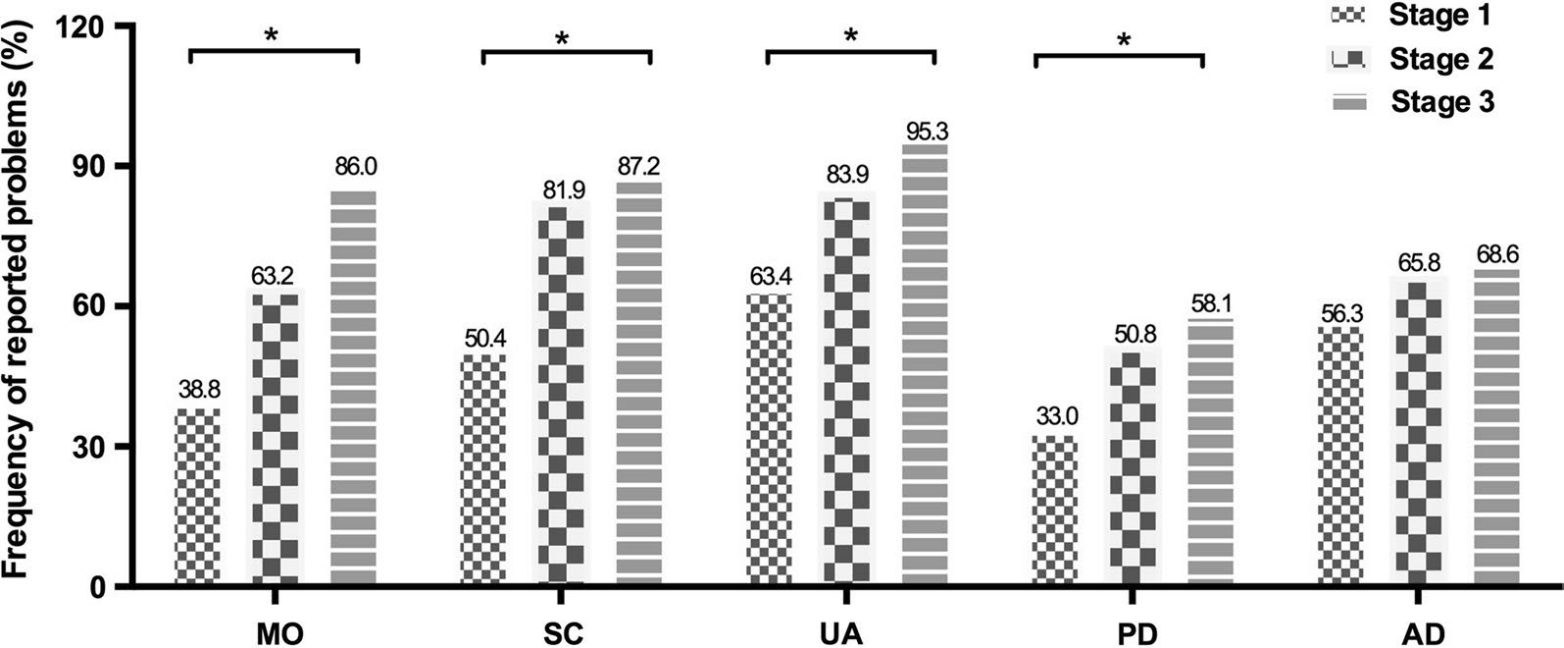
The proportion of reported problems for each of the EQ-5D-5L dimensions in different ALS stages

The median health utility score was 0.78 and the median EQ-VAS score was 70 for all 503 ALS patients. Seven patients had negative health utility scores, and 33 patients reported full health utility scores. It was convenient to dichotomize the EQ-5D-5L levels into 'no problems' (i.e. level 1) and 'problems' (i.e. levels 2 to 5), which changed the profiles into frequencies of reported problems. The frequencies of reported problems in EQ-5D-5L are reported in Fig. 1. ALS patients had the highest frequency of reported problems in usual activities (76.7%), followed by self-care (68.8%) and anxiety/depression (62.0%). The lowest frequency (44.1%) of reported problems was with pain/discomfort (dimension 4). Figure 2 shows the sums of the proportion of reported problems for each of the EQ-5D-5L dimensions with regards to different King’s College Stages. A higher proportion of reported problems in the MO, SC, UA, and PD dimensions were observed in the later stages than in the early stages. The proportion of reported problems in the anxiety/depression dimension showed no significant differences among the different stages.

Comparisons of health utility scores and EQ-VAS scores (for demographic, motor, and non-motor symptoms) were performed between different groups (Tabe 2). Patients with late-onset, lower limb onset, lower ALSFRS-R score, higher King’s College stages, lower FAB scores, presence of depression, and anxiety had lower health utility scores and EQ-VAS scores. Patients with late-onset ALS or poor sleep had lower health utility scores, with no differences in EQ-VAS scores. Patients with EDS and RBD had lower EQ-VAS scores, but no significant differences in health utility scores (Table 2).

**Table 2:** Characteristics of health utility scores and EQ-VAS scores in different subgroups

	Groups	Number	Health utility score Median, (IQR)	*P* value	EQ-VAS score Median, (IQR)	*P* value
Age of onset	> 45	390	0.74 (0.57, 0.88)	0.026*	70.0 (50.0, 80.0)	0.542
	< 45	113	0.80 (0.63, 0.91)		70.0 (60.0, 80.0)	
Sex	Male	319	0.78 (0.58, 0.91)	0.055	70.0 (50.0, 80.0)	0.221
	female	184	0.73 (0.57, 0.87)		70.0 (50.0, 80.0)	
Onset region	Bulbar onset	62	0.90 (0.80, 1.00)	< 0.001*	70.0 (60.0, 80.0)	0.019*
	Spinal onset	441	0.73 (0.56, 0.86)		70.0 (50.0, 80.0)	
ALSFRS-R score	≥ 40	378	0.80 (0.67, 0.91)	< 0.001*	70.0 (60.0, 80.0)	< 0.001*
	< 40	125	0.53 (0.31, 0.68)		55.0 (50.0, 70.0)	
Stages	1	224	0.85 (0.69, 0.93)	< 0.001*	70.0 (60.0, 80.0)	< 0.001*
	2	193	0.69 (0.51, 0.84)		65.0 (50.0, 80.0)	
	3	86	0.66 (0.44, 0.79)		60.0 (50.0, 70.0)	
FAB	≥ 16	335	0.78 (0.59, 0.91)	0.017*	70.0 (55.0, 80.0)	0.026*
	< 16	168	0.73 (0.56, 0.86)		60.0 (50.0, 80.0)	
ACE-R	≥ 75	332	0.78 (0.59, 0.89)	0.055	70.0 (51.2, 80.0)	0.097
	< 75	171	0.73 (0.54, 0.88)		60.0 (50.0, 80.0)	
Depression	> 7	238	0.65 (0.46, 0.78)	< 0.001*	60.0 (50.0, 70.0)	< 0.001*
	≤ 7	265	0.85 (0.73, 0.93)		75.0 (60.0, 80.0)	
Anxiety	> 7	150	0.63 (0.44, 0.74)	< 0.001*	60.0 (50.0, 70.0)	< 0.001*
	≤ 7	353	0.80 (0.65, 0.91)		70.0 (60.0, 80.0)	
EDS	≥ 10	78	0.78 (0.58, 0.86)	0.663	60.0 (50.0, 80.0)	0.001*
	< 10	425	0.77 (0.57, 0.89)		70.0 (57.5, 80.0)	
RBD	≥ 5	31	0.69 (0.54, 0.84)	0.215	60.0 (50.0, 70.0)	0.033*
	< 5	472	0.78 (0.58, 0.89)		70.0 (50.0, 80.0)	
PSQI	> 5	183	0.71 (0.55, 0.85)	0.001*	65.0 (50.0, 80.0)	0.244
	≤ 5	320	0.78 (0.60, 0.91)		70.0 (51.3, 80.0)	

*Significant difference

IQR, interquartile range; VAS, visual analog scale; B, bulbar onset; UL, upper limb; LL, lower limb; ALSFRS-R, Amyotrophic Lateral Sclerosis Functional Rating Scale-Revised; FAB, frontal assessment battery; ACE-R, Addenbrooke’s Cognitive Examination-Revised; EDS, excessive daytime sleepiness; RBD, rapid eye movement sleep behavioral disorder; PSQI, Pittsburgh Sleep Quality Index

Spearman’s correlation analyses showed that the index values of ALS patients were associated with ALSFRS-R scores (r = 0.666, *P* < 0.001), HDRS scores (r = − 0.539, *P* < 0.001), HARS scores (r = − 0.464, *P* < 0.001), ACE-R scores (r = 0.120, *P* = 0.005), PSQI scores (r = − 0.197, *P* < 0.001) and RBD scores (r = − 0.117, *P* = 0.007). The EQ-VAS scores were associated with ALSFRS-R scores (r = 0.421, *P* < 0.001), HDRS scores (r = − 0.387, *P* < 0.001), HARS scores (r = − 0.314, *P* < 0.001), ACE-R scores (r = 0.127, *P* = 0.003), PSQI scores (r = − 0.102, *P* = 0.017), and ESS scores (r = − 0.144, *P* = 0.001).

Potential determinants of HRQoL using the EQ-5D-5L metric are presented in Table 3. We found that ALSFRS-R scores, depression, and anxiety were associated with healthy utility scores in all ALS patients from multivariate logistic regression analyses. Disease stages, ALSFRS-R scores, and depression were associated with EQ-VAS scores, after adjusting for other parameters.

**Table 3:** Multivariate logistic regression analysis of healthy utility scores and EQ-VAS scores for ALS patients

		Variables	Rating	*P*	OR value	Adjusted OR value	95% CI
All patients	Healthy utility score	ALSFRS-R groups	0 = ≥ 40; 1 = < 40	< 0.001	8.342	6.870	3.915, 12.054
		Depression	0 = no; 1 = yes	< 0.001	7.191	4.630	2.858, 7.501
		Anxiety	0 = no; 1 = yes	0.015	5.509	1.986	1.145, 3.442
	EQ-VAS score	Stages	0 = stage 1; 1 = stage 2; 2 = stage 3	0.042	1.879	1.353	1.011, 1.810
		ALSFRS-R groups	0 = ≥ 40; 1 = < 40	0.002	3.404	2.170	1.315, 3.582
		Depression	0 = no; 1 = yes	< 0.001	3.144	2.566	1.756, 3.748

VAS, visual analog scale; ALSFRS-R, Amyotrophic Lateral Sclerosis Functional Rating Scale– Revised; OR, odds ratio; CI, confidence interval

## Discussion

The current study is the first to explore the HRQoL profile of ALS patients in a Chinese population across different ALS stages using the EQ-5D-5L scale. Our study showed that EQ-5D-5L health utility score and EQ-VAS score were related to motor and non-motor symptoms, including age, onset region, ALSFRS-R score, King’s College clinical stage, depression, and anxiety.

In our cohort, ALS patients had the highest reported problems with usual activities (76.7%) followed by self-care (68.8%) and anxiety/depression (62.0%), which was consistent with previous studies assessing HRQoL in ALS [9, 13, 23]. Previous studies found the greatest level of impairment on the dimension of usual activities, showing how disabling ALS can be [9, 24]. As disease progressed (from stage 1 to stage 3), patients in our study reported more problems in the MO, SC, UA, and PD dimensions, but not in the anxiety/depression dimension, suggesting that HRQoL progressively declined in tandem with progressive impairment of physical function. Thus, our findings also support the notion that measuring HRQoL using the EQ-5D-5L scale is practical for clinical trials [13, 25, 26]. According to the Chinese general population value sets [8], our ALS patients had median healthy utility score of 0.78, and a median health status EQ-VAS score of 70. Our patients’ median health EQ-VAS scores of was higher than in a previous study, which had ALS patients with median EQ-VAS scores of 55 [12]. This discrepancy may be the result of older age of onset, longer disease duration, and lower ALSFRS-R scores in those patients than in our cohort. The healthy utility index and EQ-5D VAS scores in our sample were lower than those of the general Chinese population from urban [mean: 0.957 ± 0.069, and 86.0 ± 11.4), respectively] [27]. Another study indicated that the mean healthy utility index and EQ-VAS scores in ALS patients were lower than the scores reported in myasthenia gravis patients [9]. Further research on comparisons between ALS patients and other neurological disease patients is needed.

The findings from the EQ-5D-5L descriptive system highlighted that HRQoL is complex in ALS patients, particularly for different subgroups. A previous study analyzed the individual dimensions of the EQ-5D-5L measures in ALS patients and found that disease severity had a greater impact on physical than mental health [13]. Thus, we analyzed the impact of clinical factors on the health utility and EQ-VAS scores in Chinese ALS patients. Besides the severity of the disease (as assessed by the ALSFRS-R or King’s College staging systems), our univariate analysis study also found that the onset region can modulate health utility score ( i.e., patients with bulbar onset had higher scores than patients with spinal onset). A previous study also found that bulbar onset rather than bulbar impairment (as defined by ALSFRS-R bulbar scores,) had an impact on both health utility and EQ-VAS scores [12]. In addition, patients with lower limb onset had poorer HRQoL than patients with upper limb onset, possibly because this had a larger impact on mobility in daily life.

Varying degrees of mood disturbances are commonly reported by ALS patients. In our cohorts, as disease progression (from stage 1 to stage 3), patients reported more problems in anxiety/depression dimension, but did not report progressive aggravation accompanying progressive impairment of physical function. A previous systematic review showed that higher levels of anxiety and depression are related to poorer HRQoL [14]. Our study also found that patients with high depression or anxiety scores had lower healthy utility scores and EQ-VAS scores than patients with lower depression or anxiety scores. Mood disturbance had an impact on the HRQoL no matter the ALS stage. Thus management of non-motor features, such as depression, should be taken into consideration for ALS patients throughout disease progression.

Cognitive decline is also a common ALS symptom. A previous study found that the presence of dementia was not a significant predictor of HRQoL (as measured by the ALS Assessment Questionnaire-40) [28]. Another recent study found that cognitive deficits have a limited influence on HRQoL in ALS [29]. Similarly, our study also found that there was no significant impact of cognition on HRQoL in ALS, although univariate analysis showed that patients with higher FAB scores had higher health utility scores and EQ-VAS scores than patients with lower FAB scores. Sleep disturbances, such as EDS and RBD, are very common in ALS patients. However, the impact of EDS and RBD on the HRQoL in ALS patients remains unknown. Previous studies have found that sleep and sleep disturbances play an important role in both HRQoL and psychological health [30]. Patients with poor sleep had lower healthy utility scores, and patients with EDS and RBD had lower EQ-VAS scores. However, our multivariate analysis found sleep disturbances had no significant impact on HRQoL in ALS patients, after adjusting for other motor and non-motor symptoms. Given the influence of psychological factors (depression and anxiety), it was remarkable that cognitive decline or sleep disturbances were not meaningfully associated with HRQoL. It is possible that the impact of these factors was obscured in the multivariate analysis due to conjunctive correlations between mood and cognition or sleep disturbances [30, 31]. The integrated management of non-motor symptoms may substantially improve HRQoL in ALS patients.

Here, we conducted an explorative study into the HRQoL of ALS patients. However, several limitations of the present study should be noted. First, our study is a cross-sectional study. We only explored the features of the EQ-5D-5L scale and the correlations between EQ-5D-5L and clinical factors, not the longitudinal health value changes in individual patients over time. Second, the exclusion of stage 4 patients will also likely affected the distribution of EQ-5D-5L scores. We did not explore the influence of other factors that contribute to HRQoL, such as fatigue, apathy, or other comorbidities. Comparisons between available HRQoL-studies are complicated by differences in study design and the HRQoL instruments used. A single scale cannot fulfill the requirements of practicality, reliability, and validity. Future studies should demonstrate health state values with the help of various approaches and measurements [24].

## Conclusions

This study provided data on the HRQoL of ALS patients in a Chinese population assessed using the EQ-5D-5L scale across different stages. Our findings showed that it was useful in clinical practice. Variable clinical and non-motor symptoms corresponded to differences in the EQ-5D-5L health utility, EQ-VAS scores, and individual EQ-5D-5L dimensions.

## Supplementary Information

**Additional file 1: Table 1**. A brief introduction of the scales used in the present study.

## Data Availability

The datasets used and/or analysed during the current study are available from the corresponding author on reasonable request.

## Abbreviations

HRQoL: Health-related quality of life
EQ-5D-5L: EuroQol-5 dimensions
ALS: Amyotrophic lateral sclerosis
VAS: Visual analog scale
ALSFRS-R: ALS Functional Rating Scale-Revised
FAB: Frontal assessment battery
ACE-R: Addenbrooke’s Cognitive Examination-revised
HDRS: Hamilton Depression Rating Scale
HARS: Hamilton Anxiety Rating Scale
PSQI: Pittsburgh Sleep Quality Index
ESS: Epworth Sleepiness Scale
RBDSQ: Rapid eye movement sleep behavior disorder Screening Questionnaire
EDS: Excessive daytime sleepiness
RBD: Rapid eye movement sleep behavioral disorder
HS: Health status
