# Supplementary material for: Health-related quality of life in amyotrophic lateral sclerosis using EQ-5D-5L
Source: Health Qual Life Outcomes. 2021 Jul 20;19:181. doi: 10.1186/s12955-021-01822-9 (PMC8290546; doi:10.1186/s12955-021-01822-9)
Supplement: Supplementary file 1 — Additional file 1: Table 1. A brief introduction of the scales used in the present study. [file 12955_2021_1822_MOESM1_ESM.docx]

Supplementary Table 1. A brief introduction of the scales used in the present study.

| Scales | Domains | Score range | Clinical significance |
| --- | --- | --- | --- |
| ALSFRS-R | 12 domains. | 0 to 4 for each domain.  0 to 48 for total score. | Lower ALSFRS-R scores indicate worse severity of the disease. |
| EQ-5D-5L | Part 1: 5 dimensions.  Part 2: A calibrated visual analog scale | Part 1: The scores can be converted into a single aggregated “health utility” score anchored at 1 (perfect health) and 0 (death).  Part 2: 0 to 100. | Higher scores indicate better health-related quality of life. |
| FAB | 6 domains. | 0 to 3 for each domain.  0 to 18 for total score. | Higher FAB scores indicate better frontal lobe function. |
| ACE-R | 5 cognitive domains. | 0 to 100 for total score. | ACE-R score <75 was defined as cognitive dysfunction according to our previous study. |
| HDRS | 24 items | 0 to 2 or 0 to 4 for each item.  0 to 76 for total score. | HDRS scores >7 indicated depression. |
| HARS | 14 items | 0 to 4 for each item.  0 to 56 for total score. | HARS scores >7 indicated anxiety. |
| PSQI | 19 self-rated questions and 5 questions rated by the bedpartner or roommate. | 0 to 3 for each component item.  0 to 21 for total score. | PSQI scores >5 indicated poor sleep quality. |
| ESS | 14 items | 0 to 3 for each item.  0 to 24 for total score. | Excessive daytime sleepiness was diagnosed by a total ESS score of ≥10. |
| RBDSQ | 10 items | 0 to 1 for each item.  0 to 13 for total score. | RBD was defined by an RBDSQ score of ≥5. |

Abbreviations: ALSFRS-R = Amyotrophic Lateral Sclerosis Functional Rating Scale– Revised; FAB = Frontal assessment battery; ACE-R = Chinese version of Addenbrooke’s Cognitive Examination-revised; HDRS = Hamilton Depression Rating Scale; HARS = Hamilton Anxiety Rating Scale; PSQI = Pittsburgh Sleep Quality Index; ESS = Epworth Sleepiness Scale; RBD = Rapid eye movement sleep behavior disorder Screening Questionnaire; EQ-5D-5L = Five-level EuroQol-5 dimensions.
